# Relationship between growth mindset and competitive motivation: a moderated parallel mediation model and feature importance analysis

**DOI:** 10.3389/fpsyg.2025.1576649

**Published:** 2025-05-23

**Authors:** Junchen Deng, Zhipeng Wang, Haiwei Chen, Bo Song

**Affiliations:** College of Sport Arts, Guangzhou Sport University, Guangzhou, China

**Keywords:** growth mindset, competitive motivation, elite athletes, basic psychological needs, stress response, moderated mediation, feature importance analysis

## Abstract

While growth mindset theory has been extensively studied in education, its influence on competitive motivation in sports contexts remains less understood. This study investigates how growth mindset (GM) affects competitive motivation (CM) among university athletes through stress response (SR) and basic psychological need satisfaction (BNS), with elite athlete status as a moderator. Analysis of data from 490 university athletes (250 elite, 240 non-elite) in Guangzhou revealed that GM positively relates to CM, partially mediated by reduced SR and increased BNS. Remarkably, elite athlete status demonstrated substantially stronger effects on these mediating pathways than GM itself, with elite athletes showing enhanced benefits from GM compared to non-elite peers. Feature importance analysis further identified dimension-specific predictors across different motivational aspects: autonomy most strongly predicted social recognition motivation, GM primarily influenced athletic ability improvement, while environmental factors and competition losses differentially affected entertainment and effort orientations. These findings expand GM applications in competitive sports and suggest that psychological interventions might yield stronger effects for elite athletes, highlighting the critical interplay between athletic development level and psychological factors in CM.

## 1 Introduction

Athletic achievement depends not only on physical capabilities but also on psychological factors that shape how athletes approach competition (Gould and Maynard, 2009). When athletes believe their abilities can grow substantially through dedication and learning, what psychologists term a “growth mindset,” their motivational patterns often differ markedly from those who view abilities as largely fixed traits (Dweck, 2006; Yeager and Dweck, 2020). This mindset distinction manifests in elite sports, where successful athletes frequently attribute their accomplishments to embracing challenges and learning from setbacks rather than innate talent alone (Sarkar and Fletcher, 2014; Rees et al., 2016). In competitive contexts, the difference becomes particularly apparent: athletes with growth mindsets often analyze defeats for improvement opportunities, whereas those with fixed mindsets might interpret the same losses as evidence of limited ability (Gardner et al., 2015). While research has extensively documented the benefits of growth mindset in academic contexts (Sisk et al., 2018), its role in competitive sports motivation remains inadequately explored, particularly regarding the psychological mechanisms that might explain this relationship (Slater et al., 2015).

Growth mindset is specifically defined as a belief system in which individuals perceive their abilities as malleable qualities that can be developed through effort, learning, and perseverance (Dweck, 2006). This contrasts with a fixed mindset, where abilities are viewed as largely stable traits with limited potential for development. Growth mindset theory has generated substantial research across various domains, with consistent evidence suggesting its positive association with adaptive motivational patterns, particularly in academic settings (Burnette et al., 2013).

Within the context of sports psychology, competitive motivation represents the psychological drive that energizes and directs athletic behavior toward achievement in competitive contexts (Tenenbaum and Eklund, 2007). Unlike academic motivation, competitive motivation in sports operates within environments characterized by direct performance comparison, public evaluation, and immediate feedback (Ames, 1995). Following the conceptual framework developed by Gill and Deeter (1988) and adapted by Ye et al. (1999), competitive motivation encompasses multiple dimensions including social recognition, improvement of athletic ability, entertainment, sensory experience, and effort orientation (Ye et al., 1999). The sports environment presents unique motivational challenges compared to academic settings, creating a context where the influence of mindset on motivational processes may operate differently than in classroom environments (Duda and Hall, 2001).

The connection between growth mindset and competitive motivation has begun to emerge in recent literature, though research remains limited. Studies have linked growth mindset to improved performance recovery after competitive failures (Shaffer et al., 2015), stronger performance-approach goals (Stenling et al., 2014), greater persistence during challenging training (Moles et al., 2017), and reduced competitive anxiety (Schleider and Weisz, 2018). Despite these promising findings, significant limitations persist in the current literature. Macnamara and Burgoyne (2023) meta-analyses identified methodological concerns in growth mindset research across domains, suggesting that previously established relationships may be more context-dependent than universally applicable. Additionally, few studies have systematically investigated the mechanisms connecting growth mindset to motivation specifically within competitive sports contexts, leaving a critical gap in our understanding of this relationship (Vella et al., 2016). The present study aims to address these gaps by examining whether growth mindset significantly influences competitive motivation among university athletes, identifying the psychological mechanisms through which this relationship operates, and determining whether these effects differ between elite and non-elite athletes.

To address this gap, we examine two psychological mechanisms that potentially mediate the relationship between growth mindset and competitive motivation (Burnette et al., 2013). First, an athlete’s stress response likely plays an important mediating role. Stress response refers to cognitive, emotional, and physiological reactions to competitive challenges (Nicholls et al., 2012). Athletes with growth mindsets typically interpret competitive stressors as learning opportunities rather than threats to their identity, resulting in more adaptive stress responses (Crum et al., 2013). Research demonstrates this cognitive reframing is associated with reduced cortisol reactivity during evaluative stress (Yeager et al., 2016) and more effective coping strategies following performance setbacks (Schroder et al., 2017). These adaptive responses potentially preserve and enhance motivation in competitive settings (Fletcher and Sarkar, 2012). In the context of competitive sports, where performance pressure is inherent, how athletes process and respond to stressors may fundamentally influence their competitive drive and goal-directed behavior (Nicholls et al., 2012).

Beyond stress response, basic psychological need satisfaction, as conceptualized within Self-Determination Theory (Ryan and Deci, 2000), represents another potential mediator. According to SDT, three fundamental psychological needs underlie autonomous motivation: competence (feeling effective in one’s interactions with the environment), autonomy (experiencing volition and self-endorsement of one’s actions), and relatedness (feeling connected to others) (Ryan and Deci, 2000). Studies in educational contexts have established positive associations between growth mindset and the fulfillment of these fundamental needs (Mouratidis et al., 2017), with initial evidence suggesting similar patterns in sports (Gardner et al., 2017). Growth-oriented athletes may experience greater need satisfaction through their approach to sport participation. Their improvement focus potentially enhances feelings of competence as they recognize development through effort. Their process orientation facilitates autonomous engagement as they view setbacks as informational rather than controlling. Their constructive approach to coaching and feedback may foster stronger relationships with teammates and coaches (Dweck, 2006; Vella et al., 2016). Through enhanced need satisfaction, growth mindset may indirectly strengthen competitive motivation in athletic contexts (Adie et al., 2008).

While these mediating mechanisms help explain the general relationship between growth mindset and competitive motivation, the strength of these relationships may vary across different athlete populations (Swann et al., 2015). The impact of growth mindset on these psychological processes and subsequent motivation likely varies based on athlete status. Elite and non-elite athletes differ substantially in their competitive experiences, technical abilities, and psychological development (Swann et al., 2015). Elite athletes often employ more sophisticated psychological skills (Calmeiro et al., 2014) and experience different patterns of need satisfaction in sport participation (Reinboth and Duda, 2006). These differences may moderate how growth mindset influences both stress responses and psychological need satisfaction among athletes at different competitive levels. This consideration is particularly relevant within Chinese sports systems, where the distinction between elite and non-elite classifications follows a standardized technical level recognition system established by the General Administration of Sport of China (General Administration of Sport of China, 2024), In this system, athletes who have attained at least a second-class athlete certificate through sanctioned competitions are categorized as elite athletes. This classification determines access to specialized coaching, competition opportunities, and institutional support, creating substantial differences in the developmental environments of elite versus non-elite athletes. Such systematic differences provide a compelling context for examining how athlete status might moderate the relationship between growth mindset and competitive motivation.

**Figure 1:**
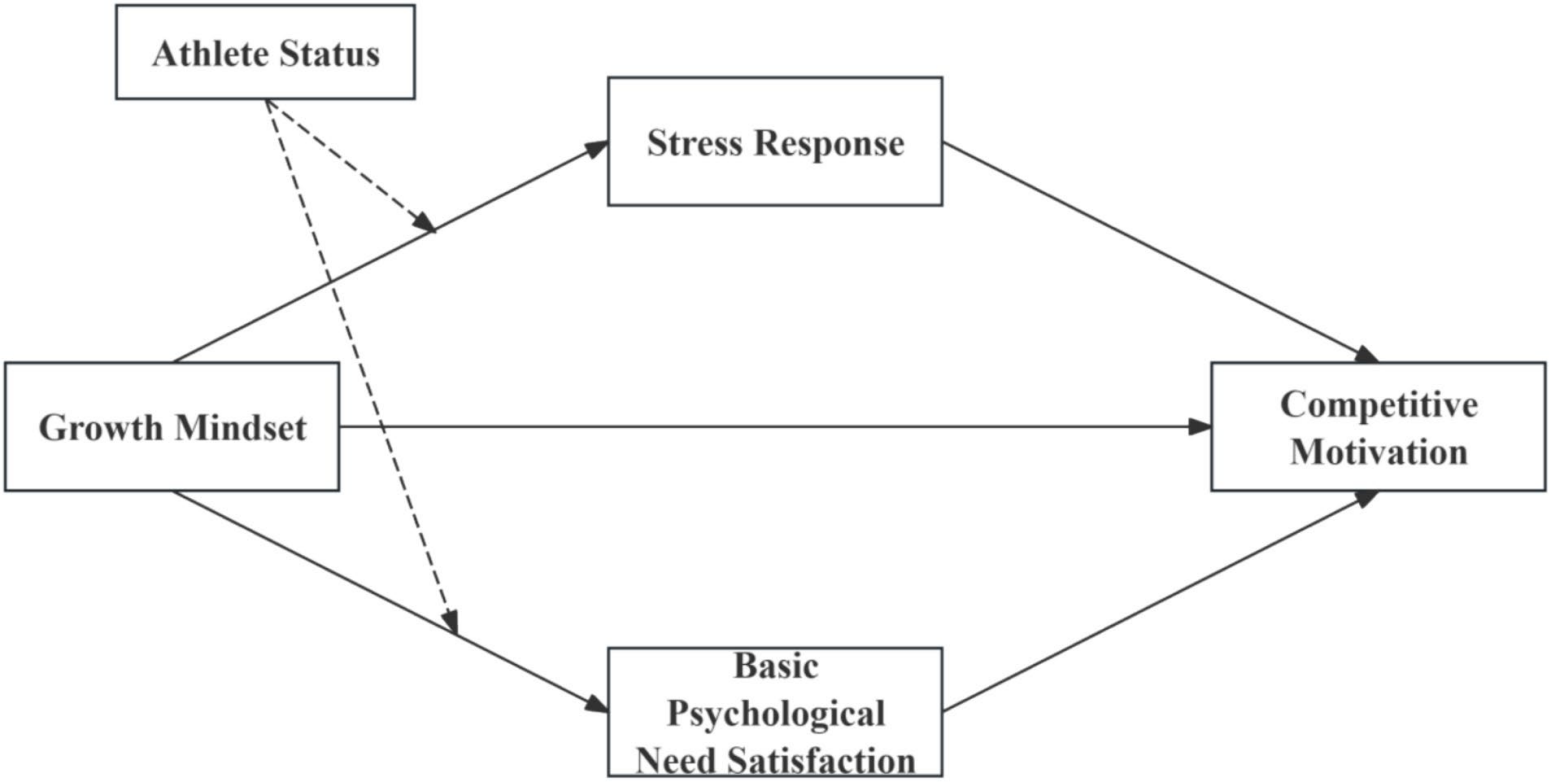
Conceptual model of growth mindset and competitive motivation. Solid lines represent direct effects and mediation pathways, while dashed lines denote moderation effects, specifically highlighting the moderating role of athlete status on the relationships between growth mindset and the mediators.

Building on these theoretical foundations and identified research gaps, the present study investigates the relationship between growth mindset and competitive motivation among Chinese university athletes through a moderated mediation model (Figure 1). This model proposes that growth mindset influences competitive motivation both directly and indirectly through stress response and basic psychological need satisfaction, with these pathways potentially varying by athlete status (Hayes, 2017). We employed both traditional statistical approaches and feature importance analysis to assess how these factors contribute to various dimensions of competitive motivation. Our approach combines ordinary least squares (OLS) regression with bootstrap analysis and machine learning techniques to provide complementary perspectives on the relationships between variables. This dual analytical approach helps address methodological concerns raised about previous growth mindset research by providing more robust examination of these relationships. Through this comprehensive investigation, we aim to deepen understanding of the psychological mechanisms connecting growth mindset to competitive motivation in sports contexts.

Based on the theoretical framework and previous research discussed above, we propose the following hypotheses:

Hypothesis 1: Growth mindset will positively predict competitive motivation among university athletes.

Hypothesis 2: The relationship between growth mindset and competitive motivation will be partially mediated by stress response, with growth mindset negatively associated with stress response, which in turn is negatively associated with competitive motivation.

Hypothesis 3: The relationship between growth mindset and competitive motivation will be partially mediated by basic psychological need satisfaction, with growth mindset positively associated with need satisfaction, which in turn is positively associated with competitive motivation.

Hypothesis 4: Athlete status will moderate the indirect effects of growth mindset on competitive motivation through both stress response and basic psychological need satisfaction, with stronger mediation effects expected for elite athletes compared to non-elite athletes.

Figure 1 illustrates the proposed conceptual model and hypothesized relationships between growth mindset, mediating variables, athlete status, and competitive motivation.

This study makes several important contributions to existing literature. First, it expands the application of growth mindset theory in competitive sports contexts, where research remains relatively limited compared to educational settings. Second, it provides empirical examination of the psychological mechanisms through which growth mindset influences competitive motivation, advancing theoretical understanding of motivation in sports. Third, by investigating elite athlete status as a moderator, it offers insights into how psychological processes may differ across varying levels of athletic development. Finally, by employing both traditional statistical approaches and feature importance analysis, this research addresses methodological concerns raised about previous growth mindset research through robust examination of these relationships.

## 2 Methods

### 2.1 Participants

This study employed a cross-sectional design with collegiate student-athletes recruited from universities in Guangzhou, China. Using convenience sampling through university athletic departments, we initially screened 1,058 athletes. After applying inclusion and exclusion criteria, the final analytical sample comprised 490 participants (250 elite athletes and 240 non-elite athletes).

Inclusion criteria for all participants were: (a) current enrollment as a student-athlete at a university in Guangzhou, (b) active participation in organized competitive sports within the past 12 months, and (c) willingness to complete all study measures. Exclusion criteria included: (a) self-reported history of significant psychological disorders that might confound questionnaire responses, (b) incomplete questionnaire responses (>10% missing data), (c) no participation in official competitive events within the past 24 months, and (d) inability to provide informed consent.

Athlete status classification followed the standardized technical level recognition system established by the General Administration of Sport of China (General Administration of Sport of China, 2024). Participants who had attained at least a second-class athlete certificate through sanctioned competitions were categorized as “elite athletes” (*n* = 250, 51.1%). Those who were active competitive athletes but had not achieved this certification level were classified as “non-elite athletes” (*n* = 240, 48.9%).

Participants’ ages ranged from 18 to 23 years (*M* = 20.09, SD = 2.67). Elite athletes reported significantly more years of competitive experience (*M* = 6.57, SD = 2.3) compared to non-elite athletes (*M* = 4.67, SD = 1.86). Detailed demographic and performance characteristics of participants are presented in Table 1, including gender distribution, competitive levels, training frequency, and years of competitive experience. The sample included athletes from diverse sporting disciplines including team sports (basketball, football, volleyball) and individual sports (badminton, tennis, gymnastics, competitive aerobics, rhythmic gymnastics, and cheerleading). Table 1 presents the participants’ characteristics.

**Table 1:** Participant characteristics.

Parameters	Categories	*N* (%) or *M* ± SD
Gender	Male	286 (58.4%)
	Female	204 (41.6%)
Athlete status	Elite Athlete	250 (51.1%)
	Non-Elite Athlete	240 (48.9%)
Highest competition level	International Competition	49 (10.00%)
	National Competition	161 (32.86%)
	Regional Competition	280 (57.14%)
Training frequency (sessions/week)	Elite Athlete	5.3 ± 1.2
	Non-Elite Athlete	3.8 ± 1.4
Competitive experience (years)	Elite Athlete	6.57 ± 2.3
	Non-Elite Athlete	4.67 ± 1.86
Age (years)	All participants	20.09 ± 2.67

### 2.2 Procedure

Data collection occurred from March to June 2023 across multiple university campuses in Guangzhou, China. The research team collaborated with athletic departments to schedule data collection sessions that minimized disruption to athletes’ training schedules. Questionnaires were administered in quiet classroom settings under standardized conditions.

Prior to participation, all athletes were informed about the study’s purpose, procedures, expected duration (approximately 25–30 min), confidentiality protections, and their rights as research participants. Participants were explicitly informed that participation was voluntary and that they could withdraw at any time without negative consequences. Written informed consent was obtained from all participants before data collection began.

Questionnaires were administered in group sessions of 10–15 athletes, with at least two research assistants present to answer questions and ensure independent completion. To minimize potential social desirability bias, coaches and team staff were not present during data collection. All questionnaires were administered in Mandarin Chinese. Participants were instructed to respond based on their general experiences in their respective sports rather than specific recent events.

All research protocols were reviewed and approved by the Institutional Review Board of Guangzhou Sport University (approval number: 2024LCLL-76), and the study was conducted in accordance with the Declaration of Helsinki regarding research involving human participants.

## 3 Measurement

### 3.1 Growth mindset

Growth mindset was assessed using the Growth Mindset Scale (GMS), which was originally developed based on Dweck’s implicit theories of intelligence (Dweck, 2006). The GMS consists of 20 items measuring beliefs about whether abilities are fixed (fixed mindset) or can be developed through effort and learning (growth mindset).

Participants responded on a 4-point Likert scale (0 = strongly disagree, 3 = strongly agree). Sample items include: “You can learn new knowledge, but you cannot really change how intelligent you are” (fixed mindset, reverse-scored) and “No matter what kind of person you are, you can always change yourself significantly” (growth mindset). The 10 items representing fixed mindset beliefs (items 1, 4, 7, 8, 11, 12, 14, 16, 17, and 20) were reverse-scored. Higher scores indicate a stronger growth mindset. The scale demonstrated good reliability in our sample (split-half reliability = 0.802).

### 3.2 Competitive motivation

Competitive motivation was assessed using the Competitive Motivation Scale (CMS) (Ye et al., 1999), a Chinese adaptation of the Sport Orientation Questionnaire (SOQ) (Gill and Deeter, 1988) developed within the framework of Achievement Goal Theory (Nicholls, 1984).

The 38-item instrument measures five dimensions: Social Recognition (8 items), Improvement of Athletic Ability (9 items), Entertainment (7 items), Sensory Experience (7 items), and Effort Orientation (7 items). Sample items include: “I enjoy being recognized for my achievements in competition” (Social Recognition); “Competition helps me improve my skills” (Improvement of Athletic Ability); “I enjoy the excitement of competition” (Entertainment); “Competition provides me with thrilling moments” (Sensory Experience); and “I put forth my maximum effort when competing” (Effort Orientation).

Items are rated on a 4-point Likert scale (1 = strongly disagree, 4 = strongly agree). Higher scores indicate stronger competitive motivation. In this study, the Cronbach’s alpha for the total scale was 0.731.

### 3.3 Stress response

Athletes’ stress responses were assessed using the Athlete Stress Scale (ASS) (Tan and Chen, 2000), which was specifically developed for Chinese athletic populations based on the Symptom Checklist-90 (SCL-90) (Derogatis et al., 1976) and the Competitive State Anxiety Inventory-2 (CSAI-2) (Cox et al., 2003).

The 45-item inventory measures six dimensions of stress response: Interpersonal Relationships (8 items), Sports Injuries (7 items), Losing Competitions (8 items), Environmental Factors (7 items), Daily Life (7 items), and Pressures (8 items). Sample items include: “I worry about my relationships with teammates” (Interpersonal Relationships); “I am concerned about getting injured during competition” (Sports Injuries); “I feel anxious about the possibility of losing” (Losing Competitions); “Unfamiliar competitive environments make me nervous” (Environmental Factors); “I have difficulty balancing training and academic responsibilities” (Daily Life); and “I feel pressured to meet others’ expectations” (Pressures).

Responses are recorded on a 5-point Likert scale (1 = not at all, 5 = extremely), with higher scores indicating greater stress response. The scale demonstrated good internal consistency in the current sample (Cronbach’s alpha = 0.751).

### 3.4 Basic psychological need satisfaction

Basic psychological need satisfaction was assessed using the Basic Psychological Need Satisfaction Scale (BPNS) (Gagné, 2003). This 21-item instrument measures satisfaction of three fundamental psychological needs: Autonomy (7 items), Competence (7 items), and Relatedness (7 items). The Chinese version of BPNS has been validated in multiple studies across Chinese populations, demonstrating good psychometric properties and cross-cultural validity (Chen et al., 2015). The scale has shown factorial invariance and similar predictive patterns of need satisfaction in both Western and Chinese contexts, supporting its applicability in Chinese athletic populations.”

Sample items include: “I feel like I am free to decide for myself how to live my life” (Autonomy), “People I know tell me I am good at what I do” (Competence), and “I get along with people I come into contact with” (Relatedness). For this study, participants were instructed to respond based on their experiences in athletic contexts.

Items are rated on a 7-point Likert scale (1 = not true at all, 7 = very true), with higher scores indicating greater satisfaction of basic psychological needs. In this study, the Cronbach’s alpha for the total scale was 0.827.

## 4 Data analysis

Data analysis was conducted using Python 3.10.9 with specialized statistical packages. After preliminary data screening and cleaning, we performed descriptive statistical analysis using the Pandas library to characterize the dataset distributions and central tendencies. Relationships between variables were examined using Pearson correlation coefficients implemented through the Scipy and Numpy libraries.

For testing the proposed parallel mediation model, we employed Ordinary Least Squares (OLS) regression via the Statsmodels library. This approach examined whether stress response and basic psychological need satisfaction statistically mediated the relationship between growth mindset and competitive motivation. All reported coefficients are standardized regression coefficients (*β*) unless otherwise specified, allowing for direct comparison of effect sizes across variables with different measurement scales. The significance of these indirect effects was assessed using bootstrap analysis with 5,000 resamples and 95% confidence intervals, which provides more robust standard error estimates without assuming normal sampling distributions.

To examine the moderating effect of athlete status, we conducted moderated mediation analysis following Hayes’ (2013) analytical framework (Model 7). This analysis tested whether athlete status (elite vs. non-elite) moderated the associations between growth mindset and both mediator variables (stress response and basic psychological need satisfaction). Simple slopes analyses were performed to interpret significant interaction effects.

To complement these traditional statistical approaches, we employed Random Forest feature importance analysis via the Scikit-learn library. The Random Forest models were constructed with the following specific parameters to ensure reproducibility: n_estimators = 500 (number of decision trees in the forest), max_depth = 10 (maximum depth of each tree), min_samples_split = 5 (minimum number of samples required to split an internal node), min_samples_leaf = 2 (minimum number of samples required to be at a leaf node), and max_features = ‘sqrt’ (number of features to consider for the best split). The criterion used for measuring the quality of splits was the Gini impurity. This machine learning technique evaluated the relative importance of 10 input features (growth mindset, six stress response dimensions, and three basic psychological need satisfaction dimensions) in predicting the five dimensions of competitive motivation. The models used a 75/25 train-test split with the Gini impurity criterion to determine feature importance. Model performance was evaluated using accuracy metrics, F1 scores, and confusion matrices to ensure the reliability of the feature importance results.

Given the cross-sectional nature of our research design, the findings represent statistical associations rather than causal relationships or temporal dynamics. All statistical tests employed a significance threshold of *p* < 0.05.

## 5 Results

### 5.1 Preliminary analyses

This study investigated the relationship between growth mindset and competitive motivation among university athletes, examining the mediating roles of stress response and basic psychological need satisfaction, as well as the moderating effect of athlete status. To address potential common method bias associated with self-reported measures, we conducted Harman’s single-factor test using Principal Component Analysis. Results revealed that the largest variance explained by a single component was 26.6%, which is considerably below the 50% threshold, indicating that common method bias did not significantly influence our findings.

### 5.2 Descriptive statistics and correlation analysis

Table 2 presents the means, standard deviations, and correlations among the main study variables. Growth mindset showed a substantial negative correlation with stress response (*r* = −0.59, *p* < 0.01) and positive correlations with basic psychological need satisfaction (*r* = 0.30, *p* < 0.01) and competitive motivation (*r* = 0.48, *p* < 0.01). Stress response was negatively correlated with both basic psychological need satisfaction (*r* = −0.50, *p* < 0.01) and competitive motivation (*r* = −0.79, *p* < 0.01). Basic psychological need satisfaction demonstrated a significant positive relationship with competitive motivation (*r* = 0.42, *p* < 0.01).

**Table 2:** Descriptive statistics and correlation analysis among variables (*N* = 490).

Variable	*M*	SD	1	2	3	4
1 GM	32.08	6.49	1			
2 SR	112.27	4.84	−0.59**	1		
3 BNS	95.80	4.65	0.30**	−0.50**	1	
4 CM	73.60	5.12	0.48**	−0.79**	0.42**	1

These variables include Growth Mindset (GM), Stress Response (SR), Basic Psychological Need Satisfaction (BNS), and Competitive Motivation (CM). Correlation coefficients indicate significant relationships among these variables, with all reported correlations significant at the **p < 0.01 level.

Additional analysis examining the relationship between athlete status and other variables revealed that elite athletes reported significantly higher levels of growth mindset [*t*(488) = 3.42, *p* < 0.01], basic psychological need satisfaction [*t*(488) = 2.86, *p* < 0.01], and competitive motivation [*t*(488) = 3.95, *p* < 0.01], as well as lower levels of stress response [*t*(488) = −3.68, *p* < 0.01] compared to non-elite athletes.

A variance inflation factor (VIF) analysis was conducted to assess potential multicollinearity among predictors. The results showed VIF values ranging from 1.28 to 2.47, with all values well below the conventional threshold of 5, indicating that multicollinearity did not substantially affect the regression analyses.

### 5.3 Direct and moderation effects

To test our conceptual model (as previously illustrated in Figure 1), we first conducted a series of regression analyses examining the direct relationships between growth mindset and our mediator variables, as well as the moderating effect of athlete status on these relationships. In the analysis predicting basic psychological need satisfaction, the model explained 14.9% of the variance [*R*^2^ = 0.149, *F*(1, 488) = 87.049, *p* < 0.01]. Growth mindset positively and significantly predicted basic psychological need satisfaction (*β* = 0.217, *t* = 7.02, *p* < 0.01). For stress response, the model accounted for 23.6% of the variance [*R*^2^ = 0.236, *F*(1, 488) = 150.86, *p* < 0.01], with growth mindset significantly and negatively predicting stress response (*β* = −0.363, *t* = −12.28, *p* < 0.01).

We next examined whether athlete status moderated the relationships between growth mindset and the mediating variables. The moderation analysis revealed significant interaction effects between growth mindset and athlete status in predicting both stress response (*β* = −0.052, *t* = −2.401, *p* = 0.017, 95% CI [−0.094, −0.010]) and basic psychological need satisfaction (*β* = 0.012, *t* = 1.123, *p* = 0.036, 95% CI [0.001, 0.023]).

**Figure 2:**
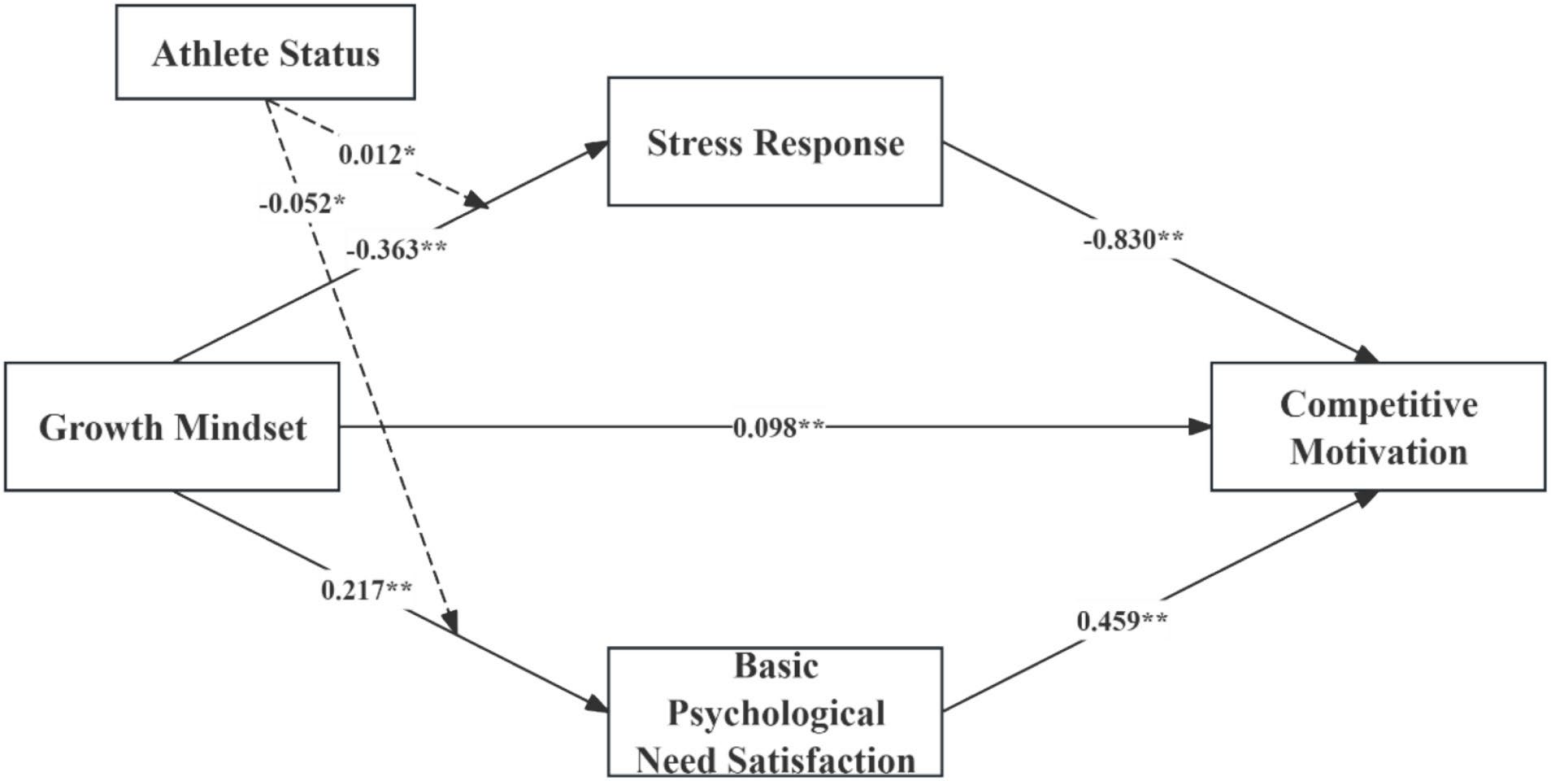
Moderated mediation model with standardized path coefficients. The diagram specifies growth mindset’s direct and indirect associations with competitive motivation, mediated through stress response and basic psychological need satisfaction, and moderated by athlete status. Significant standardized path coefficients are indicated with *p < 0.05 and **p < 0.01.

Simple slopes analysis indicated that the negative relationship between growth mindset and stress response was stronger for elite athletes (*β* = −0.219, *t* = −11.54, *p* < 0.01) compared to non-elite athletes (*β* = −0.115, *t* = −5.67, *p* < 0.01). Similarly, the positive relationship between growth mindset and basic psychological need satisfaction was stronger for elite athletes (*β* = 0.113, *t* = 6.98, *p* < 0.01) than for non-elite athletes (*β* = 0.089, *t* = 4.23, *p* < 0.01). These direct and moderation effects are presented in Table 3 and visually depicted in Figure 2, which illustrates the moderated mediation model with standardized path coefficients.

**Table 3:** Direct and moderation effects in the moderated parallel mediation analysis.

Path	*B*	S.E.	*t*	95% CI	*R* ^2^	*F*(df)
Direct effects						
GM → BNS	0.217	0.031	7.02**	[0.156, 0.278]	0.149	87.049** (1, 488)
GM → SR	−0.363	0.03	−12.28**	[−0.421, −0.305]	0.236	150.86** (1, 488)
BNS → CM	0.459	0.045	10.14**	[0.371, 0.547]	0.174	102.82** (3, 486)
SR → CM	−0.83	0.03	−28.02**	[−0.888, −0.772]	–	–
Moderation effects						
AS → BNS	1.274	0.485	2.629*	[0.323, 2.225]		
AS → SR	−1.528	0.395	−3.869**	[−2.303, −0.753]		
GM × AS → BNS	0.012	0.018	1.123*	[0.001, 0.023]		
GM × AS → SR	−0.052	0.022	−2.401*	[−0.094, −0.010]		
Simple slopes						
GM → BNS (Non-Elite)	0.089	0.021	4.23**	[0.047, 0.131]		
GM → BNS (Elite)	0.113	0.016	6.98**	[0.081, 0.145]		
GM → SR (Non-Elite)	−0.115	0.02	−5.67**	[−0.154, −0.076]		
GM → SR (Elite)	−0.219	0.019	−11.54**	[−0.256, −0.182]		

Variables examined include growth mindset (GM), basic psychological need satisfaction (BNS), stress response (SR), competitive motivation (CM), and athlete status (AS).

**Figure 3:**
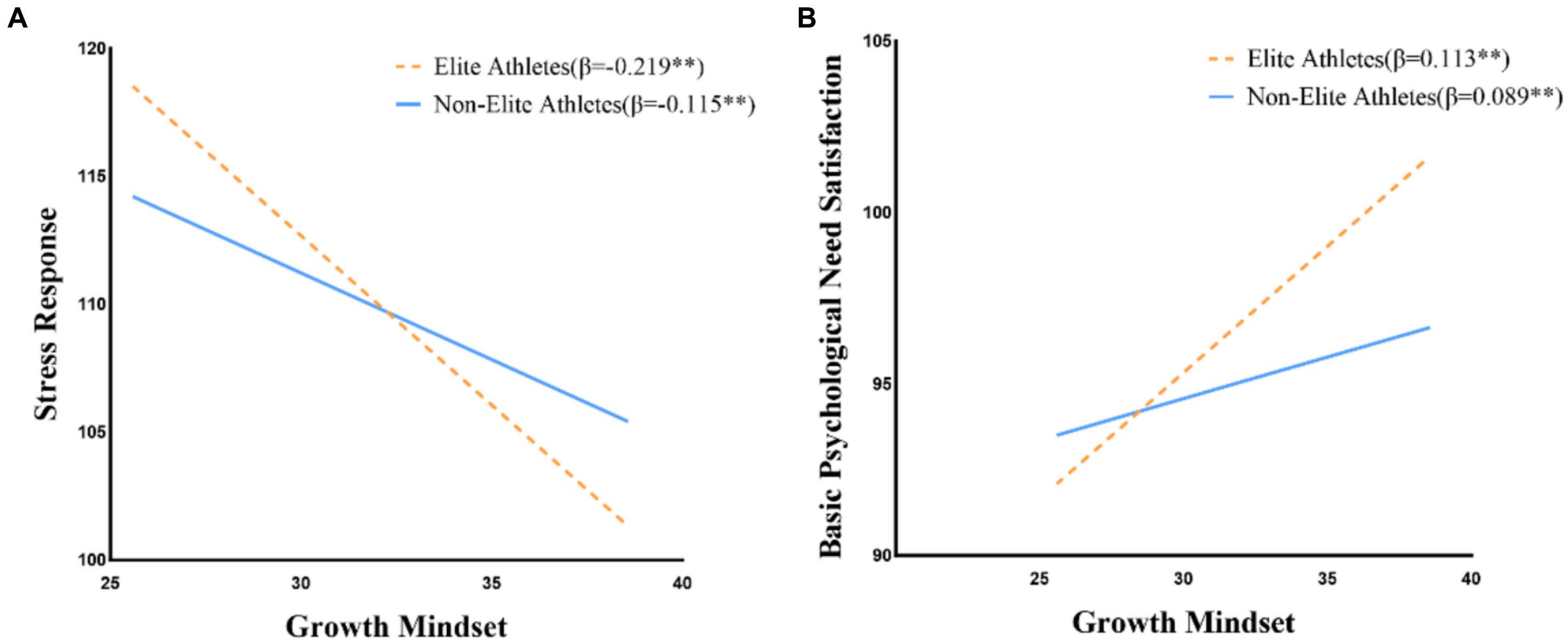
Moderation effects of athlete status on growth mindset relationships. Panel **(A)** demonstrates how athlete status moderates the relationship between growth mindset and stress response, whereas panel **(B)** highlights its moderating influence on the relationship between growth mindset and basic psychological need satisfaction.

As shown in Figure 3, the moderation effects of athlete status manifest as differences in slope steepness when plotting the relationships between growth mindset and the two mediating variables. For stress response (Figure 3A), elite athletes demonstrate a steeper negative slope (*β* = −0.219) compared to non-elite athletes (*β* = −0.115), indicating that growth mindset has a stronger stress-reducing effect for elite athletes. Similarly, for basic psychological need satisfaction (Figure 3B), the steeper positive slope for elite athletes (*β* = 0.113) compared to non-elite athletes (*β* = 0.089) suggests that growth mindset more strongly promotes need satisfaction among elite competitors.

### 5.4 Mediation and conditional indirect effects

In predicting competitive motivation, the model explained 17.4% of the variance (*R*^2^ = 0.174, *F*(3, 486) = 102.82, *p* < 0.01). As shown in Figure 2, basic psychological need satisfaction positively predicted competitive motivation (*β* = 0.459, *t* = 10.14, *p* < 0.01), while stress response negatively predicted competitive motivation (*β* = −0.830, *t* = −28.02, *p* < 0.01). Growth mindset maintained a significant direct effect on competitive motivation (*β* = 0.098, *t* = 4.99, *p* < 0.01).

To assess the significance of indirect effects, bootstrap analysis with 5,000 resamples and 95% confidence intervals was conducted. Growth mindset exhibited a significant total effect on competitive motivation (0.499, 95% CI [0.443, 0.555]). The analysis revealed two significant indirect pathways: through basic psychological need satisfaction (0.100, 95% CI [0.073, 0.131]) and through stress response (0.301, 95% CI [0.248, 0.354]). The absence of zero in both confidence intervals confirms the statistical significance of these mediating effects. These indirect effects collectively explained 80.36% of the total effect, with stress response accounting for 60.32% and basic psychological need satisfaction for 20.04%.

The direct effect of growth mindset on competitive motivation remained significant (0.098, 95% CI [0.048, 0.145]), accounting for 19.64% of the total effect. This pattern of results indicates that stress response and basic psychological need satisfaction partially mediate the relationship between growth mindset and competitive motivation, as visually represented in Figure 2.

Analysis of conditional indirect effects confirmed that the mediating effects of both stress response and basic psychological need satisfaction were stronger for elite athletes than for non-elite athletes, as presented in Table 4. The conditional indirect effect through stress response was substantially larger for elite athletes (0.182) compared to non-elite athletes (0.095), further supporting our fourth hypothesis regarding the moderating role of athlete status.

**Table 4:** Mediation and conditional indirect effects in the moderated parallel mediation analysis.

Path	Coefficient	Boot SE	*p*	95% CI	% of total effect
Mediation effects					
Total effect (GM → CM)	0.499	0.029	<0.01	[0.443, 0.555]	100
Direct effect (GM → CM)	0.098	0.025	<0.01	[0.048, 0.145]	19.64
Indirect via BNS	0.100	0.015	<0.01	[0.073, 0.131]	20.04
Indirect via SR	0.301	0.027	<0.01	[0.248, 0.354]	60.32
Conditional indirect effects					
Via BNS (Non-Elite)	0.041	0.008	<0.01	[0.026, 0.057]	
Via BNS (Elite)	0.052	0.009	<0.01	[0.035, 0.069]	
Via SR (Non-Elite)	0.095	0.017	<0.01	[0.062, 0.129]	
Via SR (Elite)	0.182	0.020	<0.01	[0.143, 0.221]	

### 5.5 Feature importance analysis

To provide additional insights beyond traditional statistical methods, we employed Random Forest feature importance analysis to evaluate the relative contributions of different predictors to competitive motivation dimensions (Breiman, 2001). For this analysis, we evaluated how 10 input features (growth mindset, six stress response dimensions, and three basic psychological need satisfaction dimensions) contributed to predicting the five dimensions of competitive motivation.

The model utilized the Gini impurity criterion (Equation 1) to determine optimal splits in the decision trees:


(1) $$\text{Gini}(p)=\sum_{k=1}^{K}\backslash \text{msapace}2{\mathrm{mup}}_{k}(1-p_{k})=1-\sum_{k=1}^{K}\backslash \text{msapace}2\mathrm{mu}p_{k}^{2}$$

where p_k represents the probability of an observation belonging to category k. Lower Gini values indicate more homogeneous nodes, with feature importance calculated by the total reduction in Gini impurity attributed to each predictor across all trees in the forest.

We employed a 75%/25% train-test split with stratification to maintain consistent class distributions. The models achieved training accuracy between 98.231 and 99.541% and test accuracy between 90.012 and 97.871%. The F1 scores (Equation 2) ranged from 0.850 to 0.970 (Chicco and Jurman, 2020):


(2) $$F1=2\times \frac{\text{Precision}\times \text{Recall}}{\text{Precision}+\text{Recall}}$$

**Figure 4:**
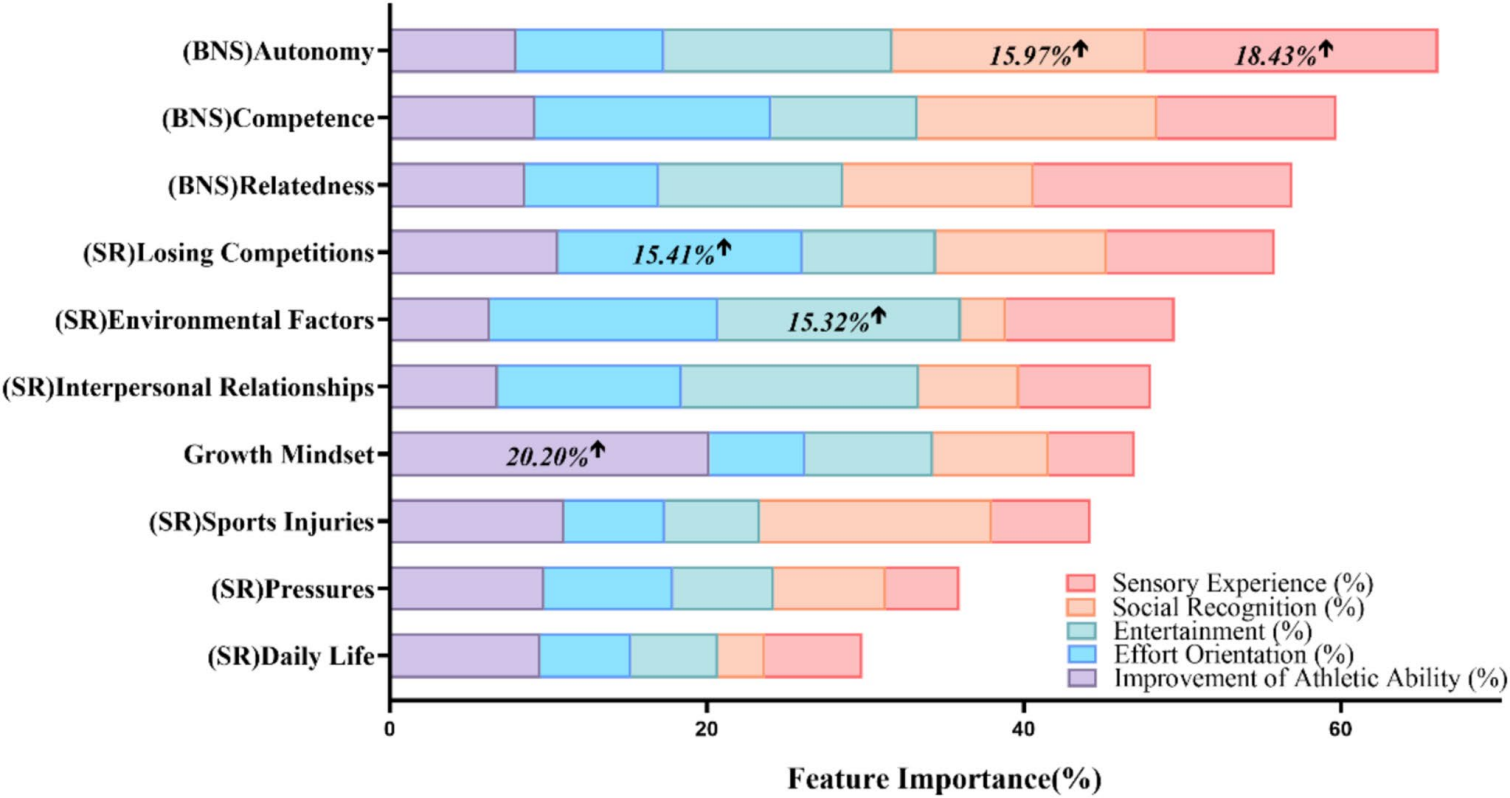
Feature importance of predictors across competitive motivation dimensions.

Figure 4 provides a visual representation of the feature importance results for each competitive motivation dimension. This visualization reveals distinct patterns of influence across different aspects of competitive motivation. As illustrated in Figure 4, autonomy (a subdimension of basic psychological need satisfaction) emerged as the most influential predictor for Social Recognition (15.97%) and Sensory Experience (18.43%). Growth mindset was the most significant feature for Athletic Ability improvement (20.20%), underscoring its particular importance for this performance-oriented dimension of motivation. For Effort Orientation, Losing Competitions (a subdimension of stress response) was most critical (15.41%), while Environmental Factors (another subdimension of stress response) was most influential (15.32%) for Entertainment.

The training accuracy of the models ranged between 98.231 and 99.541%, with test accuracy between 90.012 and 97.871% and F1 scores from 0.850 to 0.970, indicating robust predictive performance.

This feature importance analysis complements our moderated mediation results by revealing which factors most strongly predict different aspects of competitive motivation when considered simultaneously in a non-linear framework. While the mediation analysis (Figure 2) identified significant associations between growth mindset and competitive motivation through stress response and basic psychological need satisfaction, this feature importance analysis (Figure 4) provides a more granular view of how specific subdimensions contribute to different components of competitive motivation. These findings align with our hypotheses by demonstrating that both growth mindset and psychological need satisfaction contribute significantly to competitive motivation, with their effects varying across different motivational dimensions.

## 6 Discussion

This study investigated the relationship between growth mindset and competitive motivation among university athletes, examining the mediating roles of stress response and basic psychological need satisfaction, as well as the moderating effect of athlete status. Our findings provide empirical support for a moderated parallel mediation model that explains the psychological mechanisms connecting growth mindset to competitive drive within sports contexts.

Our correlation analysis revealed a substantial positive relationship between growth mindset and competitive motivation (*r* = 0.48). This finding extends previous research on growth mindset from educational contexts to the domain of competitive sports. While Macnamara and Burgoyne (2023) raised questions about the effectiveness of growth mindset interventions on academic achievement in their meta-analysis, our study advances this discourse by meticulously refining the research design, implementing rigorous data quality controls, and synergizing traditional statistical approaches with machine learning techniques. Unlike academic environments where feedback is often delayed and evaluative criteria can be ambiguous, athletic contexts provide immediate performance feedback and clear metrics for improvement, potentially creating ideal conditions for growth mindset to enhance motivation (Dweck, 2009; Vella et al., 2016).

While this direct relationship is informative, our mediation analysis revealed deeper insights into the underlying mechanisms. Stress response and basic psychological need satisfaction collectively explained 80.36% of the relationship between growth mindset and competitive motivation. Notably, stress response emerged as the stronger mediator (60.32% of the total effect) compared to basic psychological need satisfaction (20.04%). This finding differs from previous research in educational psychology, which has typically emphasized need satisfaction as the primary pathway linking growth mindset to motivation (Mouratidis et al., 2017). The prominence of stress response as a mediator suggests that how athletes cognitively frame and emotionally respond to competitive challenges may be particularly crucial in determining whether growth mindset beliefs translate into enhanced competitive motivation (Lazarus and Folkman, 1984; Fletcher and Sarkar, 2012).

This dominant mediating role of stress response aligns with the Challenge-Hindrance Stress Model (Lepine et al., 2005), which distinguishes between stressors perceived as opportunities for growth versus those perceived as obstacles to achievement. Growth mindset appears to facilitate the interpretation of competitive pressure as a challenge stressor rather than a hindrance stressor, thereby preserving motivational resources that might otherwise be depleted by excessive stress reactions. This cognitive reframing process helps explain why athletes with stronger growth mindsets maintain higher competitive motivation despite facing similar competitive pressures as their fixed-mindset counterparts (Yeager and Dweck, 2012; Crum et al., 2013).

Although less prominent than stress response, basic psychological need satisfaction still represents an important mediating pathway, encompassing the dimensions of competence, autonomy, and relatedness. Specifically, growth mindset enhances athletes’ belief in their abilities (competence), fosters interest and personal goal pursuit (autonomy), and strengthens relationships with key sports stakeholders (relatedness) (Fletcher and Sarkar, 2012; Gardner et al., 2017). These connections align with Self-Determination Theory’s proposition that the fulfillment of basic psychological needs facilitates more autonomous forms of motivation (Ryan and Deci, 2017). In competitive sports, where external pressures and comparative evaluations might otherwise undermine intrinsic motivation, growth mindset appears to help athletes maintain a sense of psychological need satisfaction that supports sustainable competitive drive (Stenling et al., 2017).

Beyond identifying these mediating pathways, our moderation results demonstrate that athlete status significantly influences the strength of these relationships. The negative association between growth mindset and stress response was substantially stronger for elite athletes (*β* = −0.219) compared to non-elite athletes (*β* = −0.115). Similarly, the relationship between growth mindset and basic psychological need satisfaction was more pronounced among elite athletes. This pattern contradicts what some theoretical perspectives might predict—namely, that growth mindset would yield greater benefits for developing athletes who have more obvious room for improvement (Dweck, 2006; Blackwell et al., 2007).

Rather than supporting this intuitive expectation, our findings align with expertise development literature suggesting that psychological factors become increasingly influential as technical competence advances (Van Mullem, 2016). This moderation effect can be interpreted in several ways. One interpretation is that elite athletes, with their more developed technical skills and competitive experience, may be better positioned to translate growth mindset into psychological benefits. This pattern might reflect elite athletes’ superior self-regulatory capabilities and more extensive repertoire of coping strategies (Keegan et al., 2014). However, alternative explanations should be considered. Selection effects may play a role, as athletes with naturally stronger connections between mindset and psychological functioning might be more likely to advance to elite levels. Additionally, the institutional environments of elite athletes often provide more sophisticated psychological support and feedback systems that could amplify the benefits of growth mindset (Henriksen et al., 2010).

These athlete status moderation findings should also be considered within the specific cultural context of our study. The Chinese sporting context, with its distinctive approach to athlete development, may create unique conditions for how growth mindset operates. Traditional Chinese cultural values emphasize effort and perseverance (Li, 2012), which align closely with growth mindset principles. Additionally, the hierarchical structure of Chinese sports training systems, where elite athletes typically train within highly structured programs that emphasize technical precision and disciplined improvement, may create conditions where growth mindset is particularly impactful for performance psychology among elite competitors (Si et al., 2011). The definition of ‘elite’ status follows specific Chinese classification standards through the athlete certification system, which may not directly translate to Western sporting contexts where athletic status is often determined differently.

Complementing these traditional statistical analyses, our feature importance analysis using the Random Forest algorithm revealed additional nuanced patterns that would not have been evident through linear analyses alone (Breiman, 2001). As depicted in our results, autonomy (a dimension of basic psychological need satisfaction) emerged as the most important feature in predicting social recognition and sensory experience aspects of competitive motivation. Growth mindset was found to be particularly influential in enhancing athletic abilities. Additionally, environmental factors (a dimension of stress response) were most impactful in relation to the entertainment attributes of competitive motivation, with losing competitions (another stress response dimension) being a leading factor in effort orientation.

These feature importance findings enhance our understanding by showing that factors influencing competitive motivation extend beyond variations in growth mindset; the roles of basic psychological need satisfaction and stress response are equally pivotal but operate differently across motivational dimensions. The high feature importance of autonomy within basic psychological need satisfaction might be attributable to the effects of autonomous experiences on social recognition. Athletes with high levels of autonomy tend to be more engaged and effective in learning and problem-solving, which contributes to greater enjoyment and recognition-seeking behavior (Clancy et al., 2017; Ryan and Deci, 2020). Similarly, the influence of environmental factors on entertainment motivation suggests that contextual elements of competition significantly shape the enjoyment athletes derive from their sport participation (Pekrun et al., 2010).

Integrating both our mediation analyses and feature importance findings with existing theoretical frameworks provides a more comprehensive understanding of motivation in competitive sports. The prominent role of stress response connects to the Challenge-Hindrance Stress Model (Lepine et al., 2005), suggesting that growth mindset may influence whether athletes perceive competitive demands as challenge stressors (potentially facilitating performance) rather than hindrance stressors (typically impairing performance). At the same time, the pattern of basic psychological need satisfaction findings aligns with Self-Determination Theory’s Basic Psychological Needs sub-theory (Ryan and Deci, 2017), which posits that satisfaction of competence, autonomy, and relatedness needs facilitates more autonomous forms of motivation. The differential prediction patterns in our feature importance analysis could be understood through the lens of SDT’s Goal Contents Theory, which distinguishes between intrinsic and extrinsic goals and their differential psychological impacts (Pekrun et al., 2010).

Translating these theoretical insights into practice, our findings have important educational implications for sport psychology practitioners, coaches, and physical education teachers. To foster growth mindset and enhance basic psychological need satisfaction among athletes, coaches should adopt autonomy-supportive coaching styles rather than controlling approaches. As demonstrated by Reeve et al. (1999), autonomy-supportive teaching methods significantly improve students’ intrinsic motivation and psychological well-being in physical education contexts. Building on this foundation, specific intervention programs have shown promise in promoting growth mindset and need satisfaction simultaneously. For instance, Mahoney et al. (2016) documented positive outcomes from a coach-delivered autonomy-supportive intervention that enhanced athletes’ basic psychological need satisfaction and reduced ill-being. Similarly, Vella et al. (2020) developed structured programs that explicitly link growth mindset principles with autonomy-supportive coaching techniques, finding that this integrated approach yields greater benefits than addressing either component in isolation. These evidence-based approaches offer practical pathways for fostering the psychological mechanisms identified in our study, ultimately enhancing competitive motivation through reduced stress responses and improved need satisfaction.

From these integrated findings, several specific practical implications emerge for athletic training and development. First, psychological interventions targeting growth mindset may be especially effective for enhancing competitive motivation among elite athletes, given the stronger mediation pathways we observed in this population (Gucciardi et al., 2015; Yeager et al., 2019). Second, the prominent mediating role of stress response indicates that stress management should be a central component of such interventions, rather than treating stress reduction and growth mindset enhancement as separate goals. Our findings suggest an integrated approach that focuses on cognitive reframing of competitive challenges to reduce their negative psychological impact (Castro-Sánchez et al., 2019).

The significant moderation effects of athlete status further suggest that growth mindset interventions should be tailored based on athletic skill level. For elite athletes, such interventions might focus specifically on applying growth mindset to performance plateaus and competitive setbacks, situations where even highly skilled athletes might default to fixed mindset thinking. These advanced interventions could incorporate visualization techniques and cognitive reframing strategies that build upon elite athletes’ existing psychological skills (MacNamara et al., 2010). For non-elite athletes, a different approach might be needed, simultaneously developing fundamental stress management skills alongside growth mindset, potentially using simpler cognitive techniques and more structured implementation strategies with greater coach guidance (Rumbold et al., 2012).

Our feature importance findings offer additional nuanced guidance for practitioners. The significant role of environmental variables in entertainment motivation suggests that sensory stimuli from the competitive environment play a crucial role in affecting athletes’ emotions and engagement. Coaches might therefore consider how training and competitive environments can be structured to enhance the entertainment value of sport participation, potentially increasing intrinsic motivation through environmental design (Gillet et al., 2010; Duda et al., 2017).

While our findings offer valuable insights, several methodological limitations warrant consideration when interpreting these results. The cross-sectional design prevents determination of causal relationships among variables, despite the statistical support for our theoretical model. While structural equation modeling provides evidence of the plausibility of the proposed relationships, experimental or longitudinal designs would be necessary to establish temporal precedence and causality (Maxwell et al., 2011). Our reliance on self-report measures introduces additional concerns about social desirability bias, which may have inflated relationships between growth mindset and motivation, as both constructs are generally viewed positively in athletic contexts. This concern is particularly relevant for elite athletes, who may face greater expectations to demonstrate positive psychological attributes.

### 6.1 Limitations

Methodological improvements in future research could include incorporating a variety of information sources, such as evaluations from coaches and peers, to enhance the reliability and validity of the data. While our Harman’s single-factor test suggested common method variance was not a major concern, multi-method approaches would further strengthen confidence in these findings. The generalizability of our results is also limited by sample characteristics. Our participants were exclusively Chinese university athletes operating within China’s distinctive sporting system, which differs considerably from Western models in terms of selection processes, training approaches, and competitive structures. The cultural emphasis on effort and perseverance in Chinese educational and sporting contexts may influence how growth mindset operates compared to more individualistic Western contexts. Additionally, the definition of “elite” status followed specific Chinese classification standards that may not directly translate to other national sporting systems.

These limitations suggest several promising directions for future research. Longitudinal studies examining how the relationship between growth mindset and competitive motivation changes over time would provide stronger evidence for the proposed relationships and reveal potential developmental patterns. Experimental studies testing targeted mindset interventions could determine whether manipulating growth mindset produces the predicted changes in stress response, basic psychological need satisfaction, and competitive motivation. Cross-cultural research comparing these relationships across different sporting cultures and systems would help determine the generalizability of our findings beyond the Chinese sporting context. Finally, more diverse methodological approaches, such as mixed-methods designs incorporating qualitative insights, could provide richer understanding of the psychological processes linking growth mindset to competitive motivation in sports.

## 7 Conclusion

This study examined the relationship between growth mindset and competitive motivation among university athletes. Our findings reveal that growth mindset positively influences competitive motivation among Chinese university student-athletes through reduced stress response and increased basic psychological need satisfaction. Elite athletes show significant advantages in these pathways compared to non-elite athletes, with elite athlete status demonstrating stronger effects on mediating variables than growth mindset itself. Feature importance analysis revealed that autonomy primarily predicts social recognition motivation; growth mindset mainly influences athletic ability improvement; environmental factors affect entertainment characteristics; and competition losses impact effort orientation. These findings expand growth mindset applications in competitive sports contexts and suggest that psychological interventions might be particularly effective for elite athletes, highlighting the critical interplay between athletic development level and psychological processes in competitive motivation.

## Data Availability

The original contributions presented in the study are included in the article/supplementary material, and further inquiries can be directed to the corresponding author.
